# Associations between the duration of outdoor sunlight exposure and the risk of developing 134 different diseases

**DOI:** 10.3389/fpubh.2026.1896657

**Published:** 2026-10-26

**Authors:** Sicheng Li, Hanwen Zhou, Wenpan Xian, Yan Wang

**Affiliations:** 1 Xiamen Cardiovascular Hospital of Xiamen University, School of Medicine, Xiamen University, Xiamen, Fujian, China; 2 School of Public Health, Chongqing Medical University, Chongqing, China; 3 Department of Stomatology, School of Medicine, The First Affiliated Hospital of Xiamen University, Xiamen University, Xiamen, Fujian, China

**Keywords:** cohort study, environmental epidemiology, inflammation and aging, public health, vitamin D, UK Biobank

## Abstract

**Background:**

Existing studies of sunlight exposure and health have mainly focused on specific organs, particularly the skin and eyes, while associations with other organs and systems remain largely unexplored. We aimed to comprehensively examine the associations between outdoor sunlight exposure duration and 134 diseases.

**Methods:**

Using UK Biobank data, summer and winter outdoor durations were collected by questionnaire, and average daily exposure was calculated as their arithmetic mean. Incident diseases were defined using 525 three-character ICD-10 codes. Cox proportional hazards models estimated associations, and restricted cubic splines assessed potential nonlinearity.

**Results:**

Among 281,282 participants [mean (SD) age, 55.81 (8.07) years; 55.79% female], 1,115,346 incident cases occurred during a median follow-up of more than 13.4 years. After false discovery rate correction, sunlight exposure was associated with 36 diseases, with hazard ratios ranging from 0.914 to 1.086. For example, each additional hour of average exposure was associated with higher risks of malignant melanoma of the skin (HR = 1.052, 95% CI 1.012–1.094), obesity (1.039, 1.027–1.052), cerebrovascular diseases (1.028, 1.011–1.046), and osteoarthritis (1.042, 1.033–1.052), and lower risks of osteoporosis (0.975, 0.957–0.994), disorders of mineral metabolism (0.968, 0.944–0.992), and pressure ulcers (0.947, 0.916–0.979). Thirty diseases, including depression, anxiety, sleep disorders, arrhythmias, iron deficiency anemia, and kidney failure, showed U-shaped associations.

**Conclusion:**

Outdoor sunlight exposure duration showed positive, inverse, and nonlinear associations with a broad range of diseases. These findings highlight the potential importance of moderate exposure and appropriate sun protection but require validation in other populations.

## 1 Introduction

Sunlight exposure is one of the most common environmental factors and exerts a dual influence on human health. On the one hand, sunlight exposure stimulates vitamin D synthesis (1), which is linked to reduced blood pressure (2, 3) and improved mental health (4). On the other hand, sunlight exposure can induce inflammation (5), aging (6), and suppression of adaptive immunity (7), further increasing the risk of developing diseases such as cataracts (8) and skin cancer (9, 10). Moreover, recent epidemiological studies have reported a U-shaped relationship between sunlight exposure and certain diseases, including depression (11), dementia (12), and Parkinson’s disease (13).

Nevertheless, two main deficiencies exist in current research on the association between sunlight exposure and the risk of developing various diseases. First, these studies predominantly focused on organs such as the skin (9, 10), eyes (8), and brain (11–13), neglecting the impacts of sunlight exposure on other systems, including the respiratory, endocrine, and digestive systems, or cancers other than skin cancer. Biologically, vitamin D does not have specific target organs (14), and the inflammation and aging induced by sunlight exposure are not confined to the skin (15, 16), suggesting that sunlight exposure may have widespread effects on multiple organs rather than being limited to specific organs. Moreover, this organ-specific focus might also stem from publication bias (17), where researchers prefer to publish studies with significant findings. Second, there are significant discrepancies in the design, sample size, covariate adjustment, outcome definition, and other aspects among existing studies (2–4, 11, 18), resulting in poor comparability. Furthermore, certain cross-sectional (3, 18) and small-sample (2, 4) studies may suffer from inadequate intensity of evidence and statistical power.

Considering that sunlight may be a double-edged sword, an outcome-wide analysis strategy is more appropriate (19). This strategy enables a comprehensive investigation of the relationships between sunlight exposure and the risk of developing various diseases across different organs and systems, promoting the prioritization of public health recommendations (19). Additionally, an outcome-wide strategy based on a large-scale cohort study ensures comparability among associations between sunlight exposure and the risk of developing different diseases while also ensuring high statistical power and strength of evidence. Thus, an outcome-wide strategy was used to investigate the associations between sunlight exposure and the risk of developing various diseases. Specifically, using data from the UK Biobank, we comprehensively investigated the associations of self-reported daily outdoor durations in summer and winter, as well as their arithmetic mean, with the risk of developing 134 diseases defined using 525 three-character ICD-10 codes.

## 2 Methods

### 2.1 Study population

This study utilized data from the UK Biobank (20). Between 2006 and 2010, the UK Biobank gathered detailed data on more than 500,000 participants, covering general characteristics, lifestyle, disease history, family history, physical examinations, and laboratory tests. During follow-up until 2022, the disease and mortality status of the participants was recorded. The UK Biobank received approval from the North West Multi-center Research Ethics Committee, with all participants providing written informed consent (21, 22).

A total of 502,178 participants from the UK Biobank were included in the study. We excluded participants with (1) a sunlight exposure duration exceeding the typical day length in the UK during summer (16 h; *n* = 252) and winter (8 h; *n* = 5,318) (23); (2) extreme sunlight exposure durations, defined as values surpassing the mean by more than twice the standard deviation, during both summer (*n* = 14,600) and winter (*n* = 24,947) (12); (3) missing sunlight exposure duration data for either summer (*n* = 33,391) or winter (*n* = 6,116); and (4) missing covariate data (*n* = 136,272). Ultimately, 281,282 participants were included in the analyses (Supplementary Figure S1). Because it was necessary to further exclude prevalent cases before the baseline survey when analyzing specific diseases, the sample size was slightly less than 281,282 and varied among the different diseases in the actual analyses.

### 2.2 Measurement of sunlight exposure

In the baseline survey, participants were asked, “In a typical day in summer (Field 1,050) and winter (Field 1,060), how many hours do you spend outdoors?”. Responses included integers or specific values such as “Do not know,” “Prefer not to answer,” or “Less than an hour a day.” The first two responses were treated as missing data, while the latter were reassigned to half an hour. Additionally, the average daily sunlight exposure duration was calculated as the arithmetic mean of summer and winter sunlight exposure durations, consistent with previous UK Biobank studies (12, 23). This measure was intended to summarize habitual outdoor duration across the two contrasting seasons rather than to estimate cumulative annual solar radiation.

### 2.3 Measurement of outcomes

To enable an outcome-wide design, all recorded diseases coded by the ICD-10 were reviewed. Chapters of the ICD-10 that were deemed irrelevant to the study population, such as pregnancy-related diseases (O00-O99), perinatal diseases (P00-P96), and congenital diseases (Q00-Q99), were excluded. Due to the limited systematic research on the health effects of sunlight exposure, the original three-character ICD-10 codes (from A00 to N99) were aggregated based on disease characteristics. Diseases with fewer than 500 incident cases were excluded to ensure adequate statistical power, given the relatively modest effect size (HR of approximately 0.9–1.1) observed in previous studies (11–13). Finally, 134 different diseases, consisting of 525 three-character ICD-10 codes, were included in this study, with a median incident case count of 5,765. Approximately 74.6% (cases >2,157) and 64.9% (cases >3,456) of these diseases could achieve 0.6 and 0.8 power, respectively, to detect an HR of 1.1 per hour of sunlight exposure at a significance threshold of *p* < 0.05.

Disease data were extracted from the “First Occurrence” section of the UK Biobank. When multiple diseases were aggregated into a single category, the earliest onset date among them was designated as the onset date for the aggregated disease. Follow-up ceased upon the earliest occurrence of disease, death, loss to follow-up, or the end of the follow-up period (2022-10-13 for England; 2022-08-31 for Scotland; and 2022-05-31 for Wales) (24). The follow-up period was calculated as the duration between the baseline survey date and the end of follow-up.

### 2.4 Statistical analyses

Cox proportional hazards regression models were used to estimate the associations between sunlight exposure and the risk of developing various diseases. Covariates included age, sex, education, employment status, skin color, Townsend Deprivation Index (TDI), location, body mass index (BMI), metabolic equivalent of task (MET), sleep duration, healthy diet index (HDI), smoking status, drinking consumption, sun protection measures, vitamin D supplementation, and family history of hypertension and diabetes. Details of these covariates and the rationale for adjusting them are provided in Supplementary Text S1. These covariates were selected *a priori* on the basis of their potential associations with both outdoor sunlight exposure and health outcomes. However, because a common adjustment set was applied across 134 disease outcomes, disease-specific residual confounding could not be completely excluded.

A restricted cubic spline (RCS) with three knots was used to validate the linear assumptions. Sensitivity analyses included the following: (1) excluding participants diagnosed with the disease within 2 years after the follow-up period began; (2) utilizing multiple imputation techniques (Supplementary Text S2) to address missing data; (3) further adjusting for available health-related environmental factors (including fine particulate matter, greenspace, and noise); (4) further adjusting for self-reported health status; (5) further adjusting for self-reported medication use for cholesterol, blood pressure, or diabetes; and (6) further adjusting for the baseline survey year. Sensitivity analyses were conducted with only one alteration made at a time compared to the primary analysis. Details of the variables in the sensitivity analyses and the rationale for conducting these analyses are provided in Supplementary Text S3.

Considering the significance (*P-*overall) and shape (*P*-nonlinear), 402 associations between three exposures and the risk of developing 134 different diseases were categorized into four classes: increasing (I), decreasing (D), U-shaped (U), and nonassociated (N). Diseases with *P*-overall >0.05 and *P*-nonlinear >0.05 were considered class N. Diseases with *P*-overall <0.05 and *P*-nonlinear >0.05 were considered class I if HR > 1 and class D if HR < 1. For nonlinear associations, an interior minimum of the fitted RCS curve was defined as the turning point. $H_{1}$ and $H_{2}$ were defined as the absolute differences in the fitted HR between the turning point and the lower and upper ends of the exposure range, respectively. Associations were classified as class U when the fitted HR increased on both sides away from the turning point and $\min(H_{1},H_{2})/\max(H_{1},H_{2})>0.5$ ; otherwise, they were classified as class I or D according to the overall direction of association. This empirical criterion was used only to facilitate descriptive classification and summary of the RCS patterns and did not affect the estimated exposure–response curves. Diseases in classes I, D, and U were aggregated into new categories for Cox regression and subsequent analyses.

Susceptible subpopulations were identified by adding interaction terms between exposure and variables of interest, known as potential effect modifiers, into the Cox model used in the primary analysis. Considering that the models in the subgroup and subsequent mediation analyses were based on a linear assumption, only diseases in classes I and D were included. Subgroups included age (≥60 years, <60 years), sex (same definition as in the covariates section if not redescribed), skin color, location (England, Scotland, and Wales), vitamin D supplementation status, sun protection status, and obesity status (BMI ≥ 30 kg/m^2^, <30 kg/m^2^).

Exploratory mediation analyses (25) (Supplementary Text S4) were conducted to examine whether selected baseline biomarkers and lifestyle factors statistically accounted for part of the observed associations between outdoor sunlight exposure and disease risk. Due to the associations between sunlight exposure and aging (6), inflammation (5), and lifestyle changes (23, 26), these variables were included as potential mediators. Taking aging indicators as an example, we included biological age (BA) (27), calculated by regressing eight blood indicators on chronological age (Supplementary Text S5); phenotypic age, derived from nine aging-related variables (Supplementary Text S5); and telomere length. We also extensively explored a wide range of blood markers. More details on the remaining mediators are provided in Supplementary Text S6. Candidate mediators were measured at baseline, and the outcomes were incident diseases recorded during follow-up. Although the mediators preceded the recorded disease diagnoses, exposure and mediator measurements were not temporally separated.

To address multiple comparisons, the Benjamini–Hochberg false discovery rate (FDR) procedure was applied separately to two prespecified families of statistical tests. FDR correction was applied jointly across the 402 primary exposure–disease association tests arising from 134 disease outcomes and three sunlight-exposure measures. In the mediation analysis, FDR correction was applied jointly across the 204 tests arising from 34 candidate mediators and six aggregated exposure–outcome associations. An FDR-adjusted *p* < 0.05 was considered statistically significant. All analyses were conducted using R 4.3.1.

## 3 Results

Among the study population of 281,282 individuals [mean (SD) age, 55.81 (8.07) years; 55.79% female], those with longer sunlight exposure durations (whether in summer or winter) were typically older, predominantly male and unemployed, with lower educational attainment, higher BMI and MET values, elevated vitamin D levels, and increased levels of aging and inflammation markers (Table 1). Descriptive analyses for sunlight exposure are shown in Supplementary Table S1 and Supplementary Figure S2. After a median follow-up period of over 13.4 years (Supplementary Table S1), 1,115,346 incident cases (514,871 in males and 600,457 in females) were identified, and 223,953 participants (99,633 males and 124,320 females) developed at least one of these 134 different diseases; hypertension had the highest incidence (16.2%), and hemolytic anemia had the lowest incidence (0.2%). The distribution of missing data and its patterns are shown in Supplementary Figure S3. The baseline characteristics of the study population changed significantly after individuals with missing data were excluded (Supplementary Table S2).

**Table 1:** The general characteristics of the study population.

Characteristic	Overall (*N* = 281,282)	Summer light, hours			Winter light, hours		
		<2 (*N* = 42,955)	2–4 (*N* = 173,033)	5–8 (*N* = 65,294)	<1 (*N* = 58,735)	1–2 (*N* = 180,771)	3–4 (*N* = 41,776)
Demographic characteristics							
Age	55.81 (8.07) *	53.41 (7.45)	55.49 (8.04)	58.21 (7.93)	53.99 (7.66)	55.87 (8.07)	58.09 (8.01)
TDI	−1.60 (2.91)	−1.39 (3.02)	−1.61 (2.90)	−1.71 (2.87)	−1.73 (2.85)	−1.61 (2.90)	−1.38 (3.05)
Sex (Female)	156,920 (55.79%) *	24,464 (56.95%)	98,501 (56.93%)	33,955 (52.00%)	36,582 (62.28%)	100,624 (55.66%)	19,714 (47.19%)
Work (Employed)	180,538 (64.18%)	35,151 (81.83%)	115,338 (66.66%)	30,049 (46.02%)	44,602 (75.94%)	115,689 (64.00%)	20,247 (48.47%)
Education (with a college or university degree)	111,767 (39.73%)	22,571 (52.55%)	70,313 (40.64%)	18,883 (28.92%)	27,645 (47.07%)	72,629 (40.18%)	11,493 (27.51%)
Location							
England	249,092 (88.56%)	38,535 (89.71%)	153,212 (88.54%)	57,345 (87.83%)	51,671 (87.97%)	160,291 (88.67%)	37,130 (88.88%)
Scotland	20,658 (7.34%)	2,930 (6.82%)	12,808 (7.40%)	4,920 (7.54%)	4,426 (7.54%)	13,273 (7.34%)	2,959 (7.08%)
Wales	11,532 (4.10%)	1,490 (3.47%)	7,013 (4.05%)	3,029 (4.64%)	2,638 (4.49%)	7,207 (3.99%)	1,687 (4.04%)
Skin color							
Black	1,296 (0.46%)	186 (0.43%)	771 (0.45%)	339 (0.52%)	273 (0.46%)	752 (0.42%)	271 (0.65%)
Brown	6,370 (2.26%)	1,198 (2.79%)	3,813 (2.20%)	1,359 (2.08%)	1,588 (2.70%)	3,671 (2.03%)	1,111 (2.66%)
Dark olive	4,888 (1.74%)	533 (1.24%)	2,808 (1.62%)	1,547 (2.37%)	870 (1.48%)	3,094 (1.71%)	924 (2.21%)
Fair	191,962 (68.25%)	29,089 (67.72%)	118,939 (68.74%)	43,934 (67.29%)	39,783 (67.73%)	123,859 (68.52%)	28,320 (67.79%)
Light olive	54,442 (19.35%)	6,980 (16.25%)	33,201 (19.19%)	14,261 (21.84%)	10,670 (18.17%)	35,447 (19.61%)	8,325 (19.93%)
Very fair	22,324 (7.94%)	4,969 (11.57%)	13,501 (7.80%)	3,854 (5.90%)	5,551 (9.45%)	13,948 (7.72%)	2,825 (6.76%)
Lifestyle							
BMI, kg/m^2^	27.09 (4.66)	27.08 (5.06)	27.03 (4.63)	27.27 (4.46)	27.27 (5.07)	26.99 (4.57)	27.29 (4.46)
MET, minutes/week	2,452.35 (2,391.01)	1,465.46 (1,659.35)	2,414.62 (2,282.67)	3,201.58 (2,793.34)	1,448.96 (1,670.03)	2,470.69 (2,263.09)	3,783.68 (3,036.52)
Sleep duration, hours	7.17 (1.04)	7.07 (1.04)	7.16 (1.01)	7.28 (1.10)	7.14 (1.06)	7.18 (1.02)	7.19 (1.11)
HDI	2.91 (1.28)	2.71 (1.29)	2.92 (1.28)	3.03 (1.27)	2.72 (1.28)	2.94 (1.28)	3.04 (1.28)
Vitamin D supplement (Yes)	11,524 (4.10%)	1,966 (4.58%)	6,974 (4.03%)	2,584 (3.96%)	2,447 (4.17%)	7,398 (4.09%)	1,679 (4.02%)
Smoke status							
Never	159,394 (56.67%)	26,526 (61.75%)	99,218 (57.34%)	33,650 (51.54%)	35,257 (60.03%)	102,280 (56.58%)	21,857 (52.32%)
Previous	96,328 (34.25%)	12,966 (30.19%)	58,417 (33.76%)	24,945 (38.20%)	18,450 (31.41%)	62,239 (34.43%)	15,639 (37.44%)
Current	25,560 (9.09%)	3,463 (8.06%)	15,398 (8.90%)	6,699 (10.26%)	5,028 (8.56%)	16,252 (8.99%)	4,280 (10.25%)
Alcohol status							
Never	18,319 (6.51%)	3,483 (8.11%)	10,826 (6.26%)	4,010 (6.14%)	4,150 (7.07%)	11,025 (6.10%)	3,144 (7.53%)
Special occasions only	28,076 (9.98%)	4,860 (11.31%)	16,779 (9.70%)	6,437 (9.86%)	6,287 (10.70%)	17,316 (9.58%)	4,473 (10.71%)
One to three times a month	30,834 (10.96%)	4,969 (11.57%)	19,110 (11.04%)	6,755 (10.35%)	6,936 (11.81%)	19,517 (10.80%)	4,381 (10.49%)
Once or twice a week	72,289 (25.70%)	10,658 (24.81%)	44,772 (25.87%)	16,859 (25.82%)	14,757 (25.12%)	46,641 (25.80%)	10,891 (26.07%)
Three or four times a week	70,074 (24.91%)	10,167 (23.67%)	43,914 (25.38%)	15,993 (24.49%)	14,292 (24.33%)	45,952 (25.42%)	9,830 (23.53%)
Daily or almost daily	61,690 (21.93%)	8,818 (20.53%)	37,632 (21.75%)	15,240 (23.34%)	12,313 (20.96%)	40,320 (22.30%)	9,057 (21.68%)
Sun protection							
Never/rarely	23,609 (8.39%)	4,261 (9.92%)	13,681 (7.91%)	5,667 (8.68%)	5,101 (8.68%)	14,215 (7.86%)	4,293 (10.28%)
Sometimes	91,145 (32.40%)	14,126 (32.89%)	56,295 (32.53%)	20,724 (31.74%)	18,667 (31.78%)	58,744 (32.50%)	13,734 (32.88%)
Always	59,399 (21.12%)	8,133 (18.93%)	35,990 (20.80%)	15,276 (23.40%)	12,053 (20.52%)	38,010 (21.03%)	9,336 (22.35%)
Most of the time	105,839 (37.63%)	15,794 (36.77%)	66,523 (38.45%)	23,522 (36.02%)	22,425 (38.18%)	69,133 (38.24%)	14,281 (34.18%)
Do not go out in sunshine	1,290 (0.46%)	641 (1.49%)	544 (0.31%)	105 (0.16%)	489 (0.83%)	669 (0.37%)	132 (0.32%)
Family history							
Family history of diabetes (Yes)	64,562 (22.95%)	10,009 (23.30%)	39,590 (22.88%)	14,963 (22.92%)	13,658 (23.25%)	40,988 (22.67%)	9,916 (23.74%)
Family history of hypertension (Yes)	143,271 (50.94%)	22,669 (52.77%)	88,477 (51.13%)	32,125 (49.20%)	30,924 (52.65%)	91,900 (50.84%)	20,447 (48.94%)
Potential mechanism indicators							
BA, year	54.38 (14.65)	51.35 (14.53)	53.93 (14.56)	57.56 (14.41)	52.30 (14.80)	54.31 (14.54)	57.59 (14.35)
Phenoage, year	50.95 (10.04)	48.55 (9.75)	50.55 (9.96)	53.58 (9.90)	49.24 (9.96)	50.92 (9.98)	53.49 (9.89)
Telomere	0.03 (1.00)	0.11 (0.99)	0.04 (0.99)	−0.05 (1.00)	0.08 (1.00)	0.03 (0.99)	−0.05 (1.00)
CRP (lg), mg/L	0.25 (1.05)	0.21 (1.09)	0.24 (1.05)	0.33 (1.03)	0.28 (1.08)	0.24 (1.05)	0.29 (1.03)
Neutrophil, 10^9^ cells/L	4.17 (1.39)	4.15 (1.40)	4.15 (1.39)	4.22 (1.39)	4.20 (1.42)	4.15 (1.38)	4.19 (1.39)
WBC, 10^9^ cells/L	6.80 (2.13)	6.77 (1.89)	6.78 (2.21)	6.88 (2.05)	6.85 (1.93)	6.78 (2.20)	6.83 (2.06)
SII	593.08 (370.51)	595.10 (437.12)	591.63 (360.77)	595.61 (347.55)	602.39 (428.66)	590.02 (353.06)	593.27 (355.77)
Calcium, mmol/L	2.38 (0.09)	2.38 (0.09)	2.38 (0.09)	2.38 (0.09)	2.38 (0.09)	2.38 (0.09)	2.38 (0.09)
Vitamin D, nmol/L	49.08 (20.99)	44.08 (20.00)	48.85 (20.80)	52.99 (21.37)	45.48 (20.70)	49.46 (20.86)	52.46 (21.26)

*Continuous variables are presented as mean (standard deviation), and discrete variables are presented as n (%). TDI: Townsend deprivation index; BMI, Body mass index; MET, Metabolic equivalent of task; HDI, Healthy diet index; BA, Biological age; CRP, C-reactive protein; SII, Systemic immune-inflammation index.

The risk of developing 36 different diseases was significantly associated with sunlight exposure after FDR correction (Figure 1 and Supplementary Table S1), with HRs ranging from 0.914 to 1.086. For example, each additional hour of average sunlight exposure was significantly associated with an increased risk of developing malignant melanoma of the skin [HR = 1.052, 95% confidence interval (CI) 1.012–1.094], obesity (1.039, 1.027–1.052), cerebrovascular diseases (1.028, 1.011–1.046), and osteoarthritis (1.042, 1.033–1.052) and a decreased risk of developing osteoporosis (0.975, 0.957–0.994), disorders of mineral metabolism (0.968, 0.944–0.992), and pressure ulcers (0.947, 0.916–0.979). The RCS results are shown in Supplementary Figures S4–S17, suggesting that sunlight exposure–disease associations are more complex than simple linear relationships, with the risk of developing 30 different diseases exhibiting U-shaped associations with sunlight exposure. Consistent results were observed for sensitivity analyses regarding the association direction, magnitude, and statistical significance (Supplementary Table S2).

**Figure 1:**
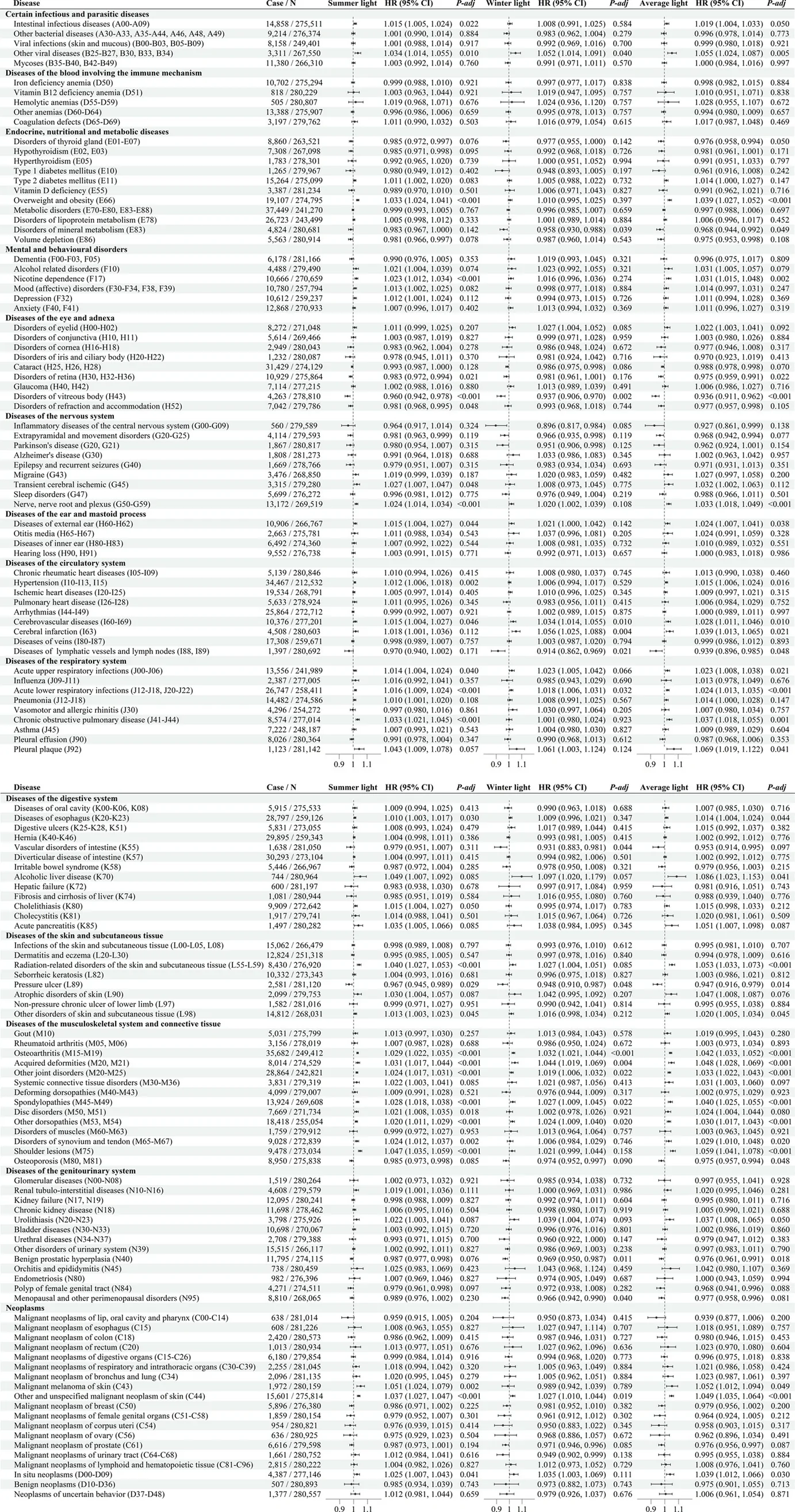
Associations between sunlight exposure and 134 diseases. All the models adjusted for age, sex, education, TDI, employment status, location, skin color, BMI, MET, HDI, sleep duration, smoking status, drinking status, sun protection, Vitamin D supplement, and family history of diabetes and hypertension. Case indicates incident cases of specific disease; N indicates the sample size of specific analysis excluding the prevalent cases, thus N is less than 281,282 and varies among diseases. HR, hazard ratio; CI, Confidence interval; *P-adj*, Adjusted *p*-value; TDI, Townsend deprivation index; BMI, Body mass index; MET, Metabolic equivalent of task; HDI, Healthy diet index.

Figure 2 illustrates some of the RCS and disease classification results. For example, summer sunlight exposure was associated with a greater risk of developing obesity and osteoarthritis. The association with the risk of developing obesity, with a *P*-nonlinear >0.05, was classified as class I. The association with the risk of developing osteoarthritis was also classified as class I, although a *P*-nonlinear <0.05 negated the assumption of linearity, but the RCS graph did not exhibit a U-shaped pattern. The results of the disease classification are shown in Table 2. In the summer sunlight exposure–disease associations, 37, 13, 13, and 71 diseases were categorized into four classes (I, D, U, and N), respectively. For example, the associations with the risk of developing three diseases of the skin and subcutaneous tissue were classified as class I, the associations with the risk of developing four endocrine, nutritional, and metabolic diseases were classified as class D, and the associations with the risk of developing four diseases of the genitourinary system were classified as class U. The associations of sunlight exposure with aggregated disease groups are shown in Figure 3.

**Figure 2:**
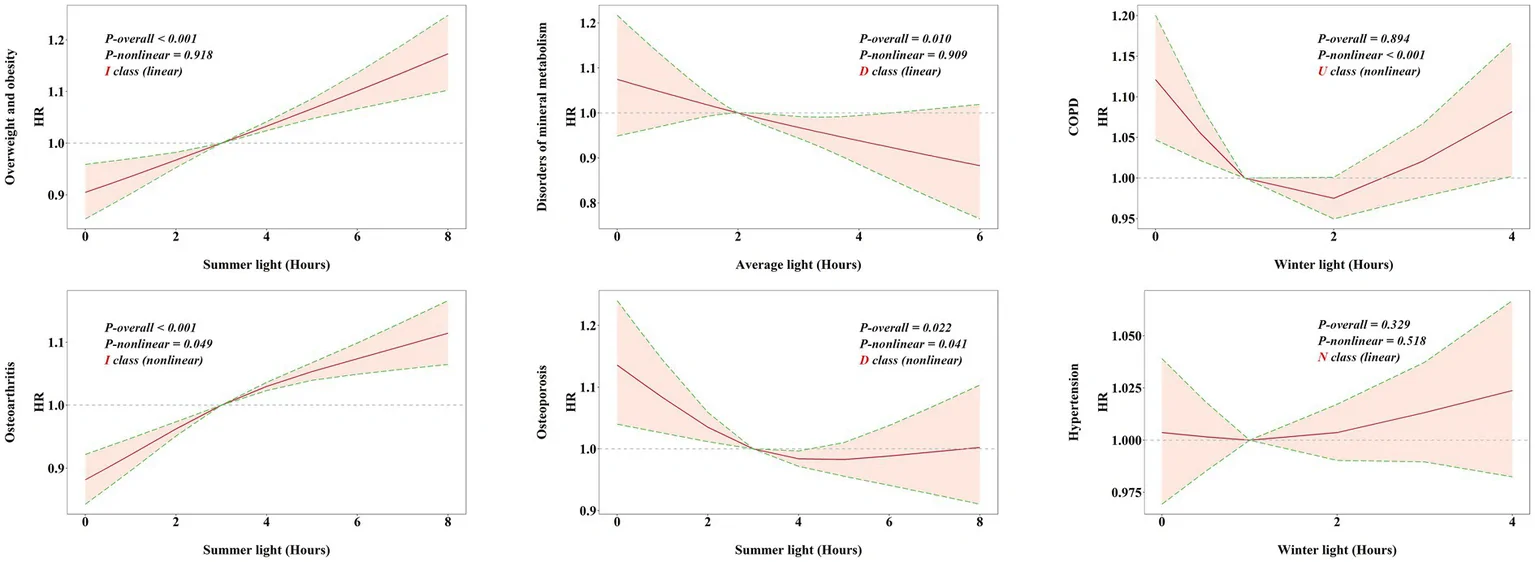
Example of classification of sunlight–disease associations. All the models adjusted for age, sex, education, TDI, employment status, location, skin color, BMI, MET, HDI, sleep duration, smoking status, drinking status, sun protection, Vitamin D supplement, and family history of diabetes and hypertension. HR, hazard ratio; TDI, Townsend deprivation index; BMI, Body mass index; MET, Metabolic equivalent of task; HDI, Healthy diet index.

**Table 2:** Summary of the sunlight–disease associations in terms of the direction and shape.

Classification of diseases (ICD-10)	No. of diseases	No. of cases	Summer light			Winter light				Average light				
			I	D *	U	N	I	D	U	N	I	D	U	N
Certain infectious and parasitic diseases	5	46,921	1	0	1	3	1	1	1	2	1	0	1	3
Diseases of the blood involving the immune mechanism	5	28,610	0	0	2	3	0	0	2	3	0	0	2	3
Endocrine, nutritional and metabolic diseases	11	131,533	2	4	0	5	0	2	2	7	1	3	0	7
Mental and behavioral disorders	6	55,592	2	0	3	1	0	0	2	4	2	0	3	1
Diseases of the eye and adnexa	9	78,844	1	4	0	4	1	2	0	6	1	4	0	4
Diseases of the nervous system	9	35,680	2	1	0	6	1	3	1	4	2	2	0	5
Diseases of the ear and mastoid process	4	29,613	1	0	0	3	1	0	0	3	1	0	0	3
Diseases of the circulatory system	9	124,226	3	0	0	6	0	1	4	4	3	1	0	5
Diseases of the respiratory system	9	86,413	4	0	1	4	2	0	4	3	4	0	0	5
Diseases of the digestive system	13	123,563	4	0	0	9	0	1	2	10	3	1	0	9
Diseases of the skin and subcutaneous tissue	8	67,722	3	1	1	3	1	1	2	4	3	1	1	3
Diseases of the musculoskeletal system and connective tissue	14	157,903	9	1	0	4	5	1	3	5	9	1	0	4
Diseases of the genitourinary system	13	89,235	2	2	4	5	1	2	4	6	1	3	2	7
Neoplasms	19	59,491	3	0	1	15	2	2 †	0	14	3	1	1	14
Total	134	1,115,346 ‡	37	13	13	71	15	16	27	75	34	17	10	73

*Based on the results of the Cox model and RCS model, the sunlight–diseases associations were classified into four main categories, namely, the increasing group (I), the decreasing group (D), the U-shaped group (U) and the no association group (N). †The association between winter light and rectum cancer approximated an inverted U-shape and could not be classified into these four categories, so results are shown here for only 18 cancers. ‡Over more than 13 years of follow-up, participants can develop multiple new diseases, so the number of cases here is much larger than the sample size of the study.

**Figure 3:**
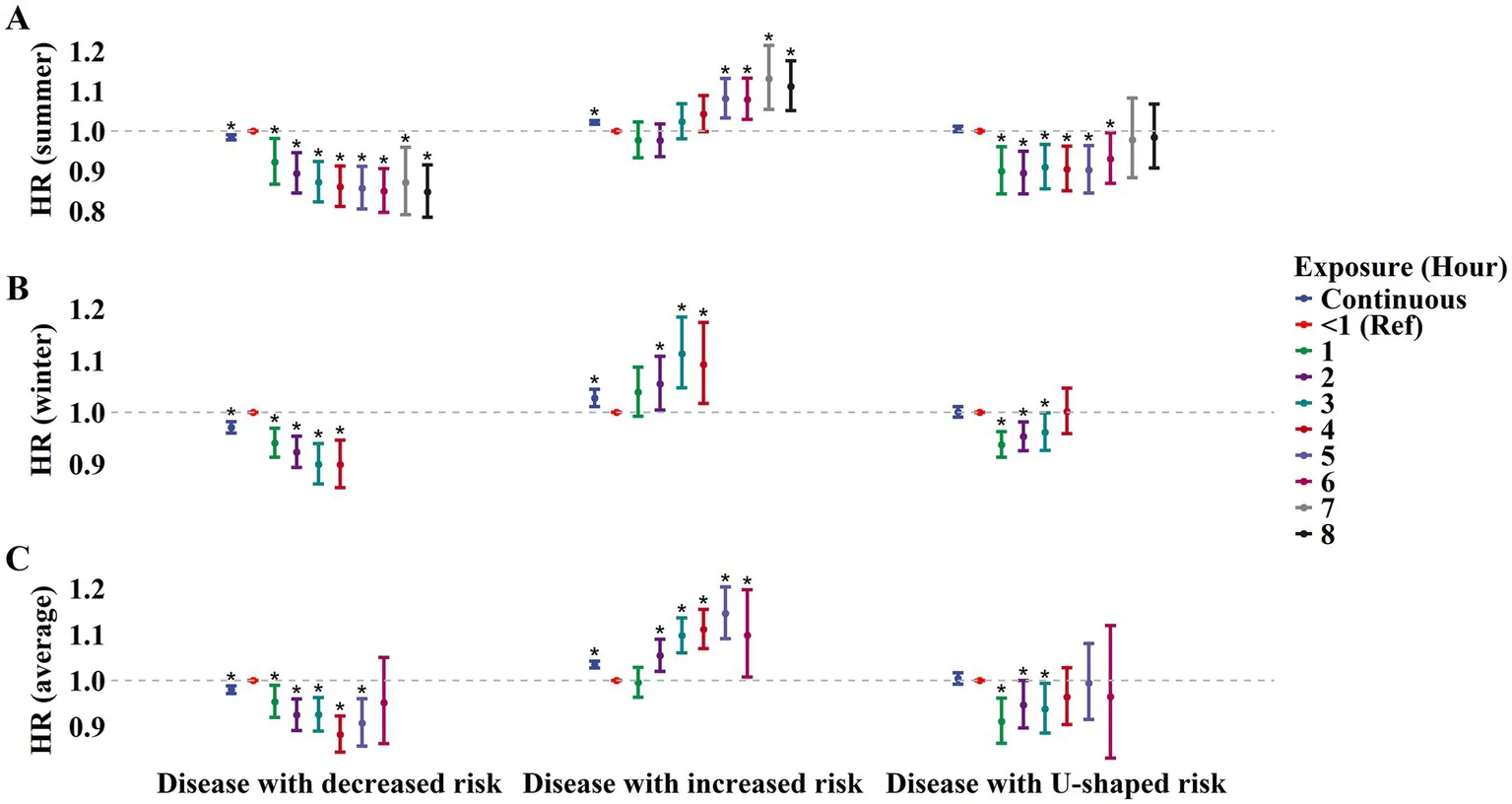
Associations of sunlight with specific classes of diseases. Panels **(A−C)** show the associations for summer, winter, and average sunlight exposure, respectively. All models were adjusted for age, sex, education, TDI, employment status, location, skin color, BMI, MET, HDI, sleep duration, smoking status, drinking status, sun protection, vitamin D supplementation, and family history of diabetes and hypertension. HR, hazard ratio; TDI, Townsend deprivation index; BMI, Body mass index; MET, Metabolic equivalent of task; HDI, Healthy diet index.

Interaction analyses indicated evidence of effect modification by age, sex, location, and BMI for some sunlight exposure–disease associations (Figure 4 and Supplementary Table S3). For the aggregated increased-risk disease group, the positive association with summer sunlight exposure was stronger among younger individuals (<60 years; P for interaction = 0.020), males (P for interaction = 0.018), and nonobese individuals (BMI < 30 kg/m^2^; P for interaction <0.001). For the aggregated decreased-risk disease group, the inverse association with summer sunlight exposure was stronger among females (P for interaction <0.001) and individuals residing in Wales (P for interaction = 0.018). For winter sunlight exposure, the positive association with the aggregated increased-risk disease group was stronger among older individuals (≥60 years; P for interaction = 0.021), males (P for interaction <0.001), and individuals residing in Wales (P for interaction = 0.045), whereas the inverse association with the aggregated decreased-risk disease group was stronger among younger individuals (<60 years; P for interaction <0.001), females (P for interaction <0.001), and individuals residing in Wales (P for interaction = 0.001).

**Figure 4:**
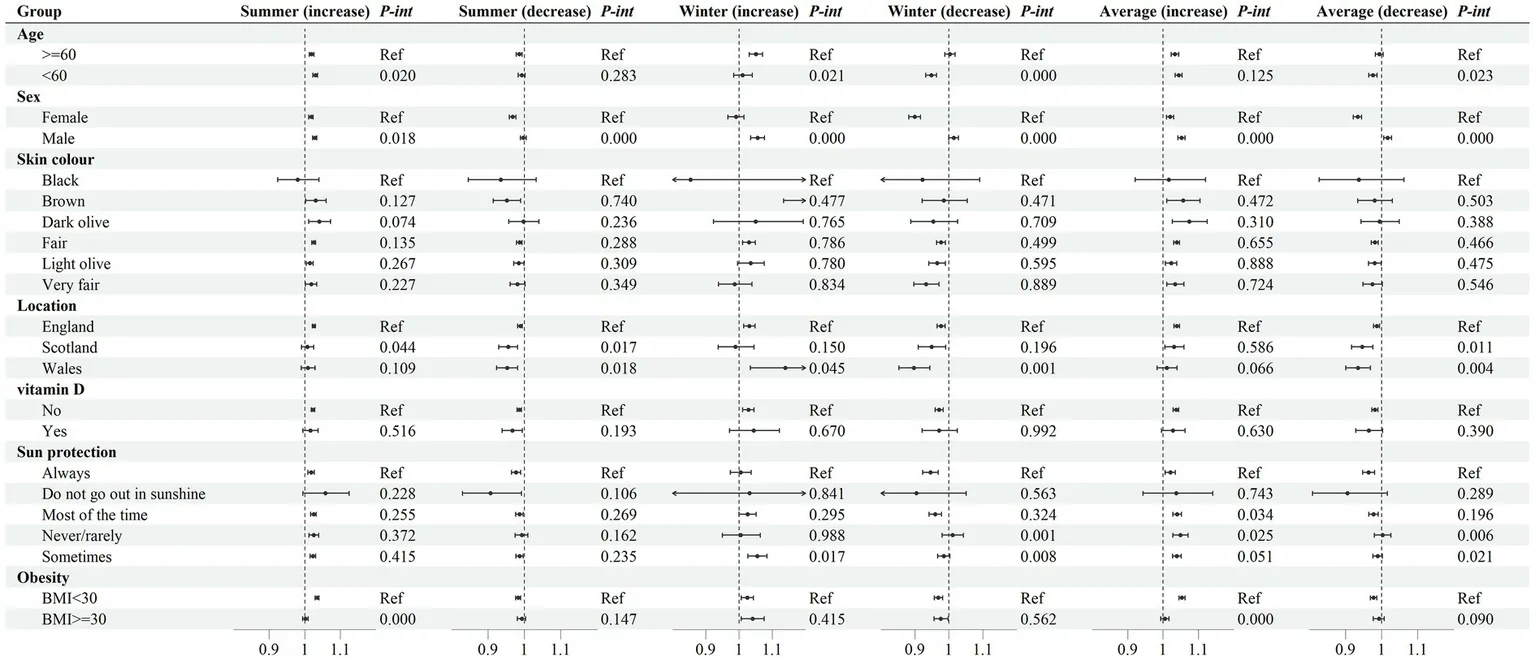
Potential effect modifiers in sunlight–disease associations. All the models adjusted for age, gender, education, TDI, employment status, location, skin color, BMI, MET, HDI, sleep duration, smoking status, drinking status, sun protection, Vitamin D supplement, and family history of diabetes and hypertension. The effect estimates given in the figure are HRs and 95% CIs. Ref, Reference; HDI, Healthy diet index; MET, Metabolic equivalent of task; BP, Blood pressure; TDI, Townsend deprivation index; BMI, Body mass index; HR, Hazard ratio; CI, Confidence interval.

Exploratory mediation analyses identified several baseline biomarkers and lifestyle factors that statistically accounted for part of the observed associations between sunlight exposure and disease risk (Figure 5 and Supplementary Table S4). For the aggregated increased-risk disease group (37 diseases), the association with summer sunlight exposure was statistically accounted for in part by BA (3.9%), neutrophil count (2.2%), BMI (18.8%), and vitamin D concentration (4.3%). For the aggregated decreased-risk disease group (13 diseases), cholesterol (2.6%), MET (27.6%), and vitamin D concentration (7.5%) statistically accounted for part of the association.

**Figure 5:**
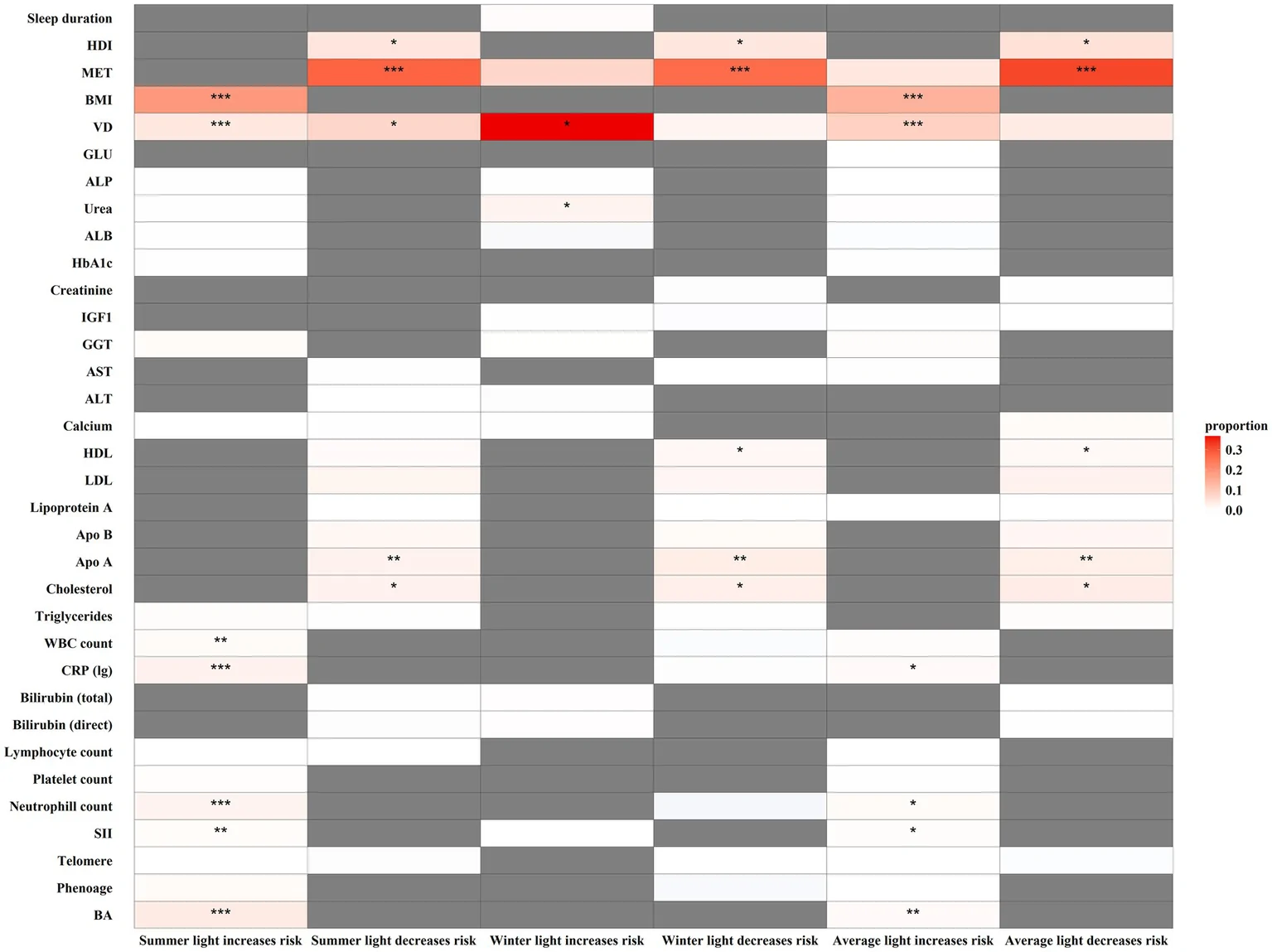
Potential mediators in the sunlight–disease associations. Both the exposure-mediator and exposure-mediator-outcome models adjusted for age, sex, education, TDI, employment status, location, skin color, BMI, MET, HDI, sleep duration, smoking status, drinking status, sun protection, Vitamin D supplement, and family history of diabetes and hypertension. When the lifestyle variable acts as a mediator, the exposure-mediator model does not control for it. Gray indicates the absence of a mediating effect (total effect and indirect effect are reversed). Red indicates a mediating effect by elevating the mediator variable, while blue indicates a mediating effect by lowering the mediator variable. The darker the color, the higher the proportion mediated. HDI, Health diet index; MET, Metabolic equivalent of task; BMI, Body mass index; VD, Vitamin D; GLU, Glucose; ALP, Alkaline phosphatase; ALB, Albumin; HBA_1c_, Hemoglobin A_1c_; IGF1, Insulin-like growth factors-1; GGT, Gamma-glutamyl transferase; AST, Aspartate aminotransferase; ALT, Alanine aminotransferase; HDL, High-density lipoprotein; LDL, Low-density lipoprotein; APO B, Apolipoprotein B; APO A, Apolipoprotein A; WBC, White blood cell; CRP, C-reactive protein; SII, Systemic immune-inflammation index; BA, biological age; TDI, Townsend deprivation index; *0.01 < *P*-adj < 0.05; **0.001 < *P*-adj < 0.01; ***P-adj < 0.001.

## 4 Discussion

To our knowledge, this is the first outcome-wide cohort study exploring associations between sunlight exposure and the risk of developing 134 different diseases. Our findings from the UK Biobank included the following: (1) considerable heterogeneity in the significance and shape of the associations between sunlight exposure and the risk of developing various diseases, with the risk of developing 36 different diseases were significantly associated with sunlight exposure after FDR correction, and the risk of developing 30 different diseases exhibiting a U-shaped association with sunlight exposure; (2) age, sex, location, and BMI modified the sunlight exposure–disease associations; and (3) aging and inflammatory markers, lipid and vitamin D concentrations, and lifestyle factors may represent potential pathways of the observed associations.

### 4.1 Comparison to other studies

Consistent with the results of previous studies, this study revealed that sunlight exposure increased the risk of developing melanoma and skin cancer (9, 10); reduced the risk of developing osteoporosis (28), Parkinson’s disease (13), and refractive disorders (29, 30); and exhibited a U-shaped relationship with the risk of developing depression (11). However, this study also revealed inconsistencies with previous research. For instance, McCarty et al., the authors of a review study, concluded that the evidence available at that time supported the association between ultraviolet radiation exposure and the risk of developing cataracts (29), which was nonsignificant in this study. This discrepancy may be due to our focus on the duration of sunlight exposure, lack of data on specific sunlight wavelengths, failure to investigate various cataract subtypes, and possibly missing covariates related to the risk of developing eye diseases, such as the use of sunglasses. Moreover, current research suggested that sunlight exposure might be associated with lower blood pressure (2, 3), whereas this study revealed a greater risk of hypertension related to sunlight exposure. For example, Weller et al. reported that in hemodialysis patients, ultraviolet intensity exposure matched by address was correlated with lower systolic blood pressure (3). This discrepancy may be due to that study being cross-sectional, involving patients rather than the general population, and using a definition of sunlight exposure that differed from the one used in the present study.

Multiple dimensions of sunlight exposure, such as intensity, wavelength, timing, and duration, are related to health (30). Among these factors, sunlight exposure intensity and duration determine the total amount of exposure (7), while wavelength (31) and timing (32) are related to the health effects of sunlight exposure. The self-reported sunlight exposure data collected in this study included only the duration of exposure. To account for intensity, we included survey location as a covariate. According to our subgroup analysis, higher-intensity sunlight exposure in Wales had stronger effects, confirming the consistency of our conclusions on the total amount of sunlight exposure. In this study, different bands of sunlight could not be distinguished due to unavailable data, but we controlled for potential reductions in ultraviolet exposure resulting from sun protection measures. Last, information on the timing of sunlight exposure was not gathered in this study. However, we mitigated the risk of participants reporting nighttime sunlight exposure due to misunderstanding by excluding those with extreme exposure durations. In summary, although this study considered only the duration of sunlight exposure, we still accounted for other dimensions of sunlight as much as possible to reduce the risk of unclear definitions of exposure. Notably, caution is still needed when comparing and extrapolating epidemiological studies using sunlight as the exposure.

### 4.2 Potential mechanisms

The association between sunlight exposure and the risk of developing skin diseases has been thoroughly investigated, with potential mechanisms involving the induction of DNA and mitochondrial damage, lipid peroxidation, and the activation of inflammatory signaling pathways (5). However, how sunlight exposure affects organs and systems beyond the skin remains unclear. From an anatomical and histological perspective, ultraviolet radiation affects the activation of Langerhans cells (antigen-presenting cells related to immune function) in the epidermis and the recruitment of immune cells in the dermis and may exert systemic effects through the rich blood vessels and leukocytes in subcutaneous tissue (7).

Some research indicates that sunlight exposure, especially ultraviolet B (UVB) exposure, promotes the synthesis of vitamin D, which in turn affects health (33). The results of the mediation analysis also suggested that the effect of sunlight exposure on health is associated with elevated vitamin D levels. Vitamin D may reduce the risk of upper-respiratory tract infections by influencing immune cells and cytokines (28, 34). However, this study revealed a positive association between those variables, which might be due to additional environmental risk factors from prolonged outdoor activities. Despite the consideration of available environmental variables in the sensitivity analyses, this approach might still be insufficient. Moreover, vitamin D can regulate mineral metabolism and reduce the risk of developing osteoporosis (34), which is consistent with our results. However, the latest review study, which involved the consolidation of numerous randomized controlled trials and Mendelian randomization studies, did not support the benefits of vitamin D supplementation for reducing the risk of developing diabetes, cancer, and cardiovascular diseases (14). In this study, in addition to the risk of developing arrhythmias, which show a U-class association with sunlight exposure, the risk of developing other cardiovascular diseases, such as hypertension, cerebrovascular disease, and cerebral infarction, all display I-class associations with sunlight exposure, further indicating the important role of potential mechanisms beyond the vitamin D concentration.

Aging, inflammation, and weight gain were suggested to play important roles in the health hazards of sunlight exposure in this study. For example, Gunn et al. reported an association between facial appearance and the risk of developing cardiovascular diseases (35). We hypothesize that inflammation caused by facial photoaging may be related to systemic inflammation (15), thereby increasing the risk of developing cardiovascular disease. If our hypothesis holds, as a component of aging (36), inflammation in the skin may be related to systemic aging (16). Regarding the association between sunlight exposure and obesity, epidemiological studies have shown that sunlight exposure is associated with a reduction in subcutaneous fat (37), an increase in visceral fat (37), and childhood obesity (38). Similarly, Kim et al. discovered that ultraviolet radiation may induce subcutaneous fat cells to produce specific chemokines, leading to subcutaneous fat loss, which could be associated with systemic inflammation and obesity (39). Parikh et al. discovered that UVB radiation increases appetite and weight gain in mice (40), particularly in males, whose skin appears more sensitive to sunlight exposure (41). For instance, male mice exhibit weaker antioxidant responses to UV radiation (42), which may explain why men are more sensitive to the harmful effects of sunlight according to subgroup analyses. Furthermore, the newly identified skin–gut axis suggests that sunlight exposure is associated with alterations in gut microbiota (43), including an increase in Proteobacteria, which is associated with low-grade inflammation and a predisposition to obesity (44), and a decrease in Bacteroidetes, which is associated with the risk of developing obesity (45, 46). Given that obesity is a significant mediator, the adverse effects of sunlight exposure on certain metabolic diseases may be diminished in individuals with obesity, which also partly explains the results of our subgroup analysis.

In addition to the vitamin D concentration, this study revealed that changes in lifestyle and blood lipids may be potential mechanisms by which sunlight exposure benefits health. Previous studies have shown that longer sunlight exposure is associated with healthier dietary habits (47) and increased physical activity (26), which is consistent with our findings. Epidemiological studies have also shown that longer sunlight exposure is related to higher HDL levels (48), which may benefit health and is consistent with our results. However, the present study revealed that sunlight exposure reduces disease risk through increased cholesterol and apolipoprotein A concentrations, which contradicts the findings of previous studies (49), although the increase in those concentrations occurring as a result of 1 h of sunlight exposure is only approximately one one-thousandth of the average concentration. We speculate that the increase in blood lipids might be related to increased appetite. Since cholesterol is vital for the normal function of the immune system (50), this could explain why sunlight exposure reduces the risk of developing central nervous system inflammation (although this association was not significant after FDR correction). Moreover, animal experiments have shown that high serum cholesterol levels can enhance the antitumor function of natural killer cells (51), potentially explaining how sunlight exposure reduces the risk of developing certain tumors, such as prostate cancer (although this association was not significant after FDR correction).

The sunlight exposure–disease associations vary between summer and winter. Sunlight exposure in summer appears to be more strongly correlated with an increased risk of developing various diseases, while in winter, sunlight exposure shows more of a U-shaped association with the risk of developing various diseases. The reasons for this variation may include sunlight intensity and environmental temperature. The sunlight intensity in winter is significantly lower than that in summer, requiring longer exposure times in winter to reach the same total exposure level as in summer. For instance, there is a U-shaped relationship between cerebrovascular diseases and sunlight exposure in winter, with the lowest risk occurring after 1 h of exposure. However, when considering seasonal variations in sunlight intensity, differences in exposure area due to clothing, and disregarding temperature differences, sunlight exposure in summer may reach the lowest risk point in a shorter time, such as half an hour. Since the reported duration of sunlight exposure has a resolution of 1 h, the summer sunlight exposure–disease association might overlook this turning point and thus be linear. In addition, temperature may also play an important role. Since the exposure in this study is sunlight exposure duration, the influence of outdoor temperature may be included in the observed effects, even after adjusting for location. This explains why the risk of developing most diseases tends to increase with longer exposure durations. Additionally, Marion et al. discovered an interaction between sunlight exposure and temperature in relation to sunburn (52). Therefore, we hypothesize that temperature, whether high in summer or low in winter, may influence the effects of sunlight exposure, contributing to the observed seasonal variations in the effects of sunlight exposure.

### 4.3 Clinical implications

This study revealed complex associations between outdoor sunlight exposure duration and the risk of developing a broad spectrum of diseases. Given the bidirectional associations observed, the findings may provide preliminary evidence for considering moderate outdoor sunlight exposure at the population level. Based on the observed exposure–response patterns, approximately 1 h of summer outdoor sunlight exposure and 1–2 h of winter exposure per day may represent preliminary population-level reference ranges rather than definitive recommendations. Similarly, disease-specific exposure durations suggested by the observed curves, such as approximately 2 h for osteoporosis, should be regarded as hypothesis-generating targets that require validation in independent studies. Given the observational nature of this study, the modest magnitude of the observed associations, and the possibility of residual confounding, these findings should not be used to prescribe individualized sunlight exposure durations. Appropriate sun protection should also be considered when interpreting potential public health implications.

### 4.4 Limitations and strengths

We acknowledge several limitations. First, self-reported sunlight exposure duration may be impacted by recall bias and a lack of measurements of other dimensions of sunlight exposure. However, we considered covariates related to sunlight intensity, timing, and wavelength, reducing the risk of unclear definitions of exposure. In addition, the approximately 1-h resolution of self-reported sunlight exposure may have obscured finer nonlinear exposure–response patterns and turning points, particularly during summer when relevant differences in exposure may occur within shorter time intervals. Second, the complete-case analysis excluded a substantial number of participants with missing data, and several baseline characteristics differed between included participants and the population before missing-data exclusion, potentially introducing selection bias and limiting generalizability. However, the multiple-imputation sensitivity analysis yielded generally consistent results, reducing but not eliminating this concern. Third, although a cohort study design was employed and participants with specific diseases at baseline were excluded, the risk of reverse causation persisted. Consistent results were obtained in sensitivity analyses where participants who developed disease within the first 2 years of the follow-up period were excluded, easing our concerns. Fourth, a single covariate control strategy was used for the risk of developing 134 different diseases, and potential unmeasured confounders may impact the results. Although we performed sensitivity analyses for numerous variables and derived similar conclusions, the risk remains. Fifth, sunlight exposure and most candidate mediators were assessed at or around baseline, preventing confirmation of the temporal sequence between exposure and mediator. Therefore, the mediation findings should be interpreted as exploratory statistical decompositions rather than evidence of causal mechanisms. Sixth, diseases were grouped according to the direction and shape of their Cox model associations for exploratory subgroup and mediation analyses. However, diseases within the same group may differ in their pathological characteristics and underlying mechanisms. Therefore, the grouped findings should be interpreted cautiously and cannot be directly extrapolated to individual diseases. Finally, the study population was drawn from the UK Biobank and consisted predominantly of participants with lighter skin colors living in the United Kingdom. Therefore, the generalizability of these findings to other geographic regions, climates, and ethnic groups is limited and requires external validation.

This study has several strengths. First, we utilized an outcome-wide strategy, incorporating 134 different diseases across 14 major ICD-10 chapters, providing comprehensive insights into the effects of sunlight exposure on health, including detrimental, protective, and nonlinear effects. Second, we employed a large-scale cohort study design, offering a high strength of evidence, and the long-term follow-up of over 13 years ensured sufficient new cases and adequate statistical power. Third, we conducted thorough statistical analyses, including sensitivity analyses to verify the robustness of the findings, subgroup analyses to identify susceptible subpopulations, and mediation analyses to explore potential mechanisms.

## 5 Conclusion

In conclusion, outdoor sunlight exposure duration showed positive, inverse, and nonlinear associations with the risk of developing a broad range of diseases, with patterns varying across seasons and population subgroups. These findings support the importance of maintaining an appropriate duration of outdoor exposure while considering environmental conditions and adequate UV protection. Further studies are needed to validate these associations before specific population-level or individualized exposure recommendations can be established.

## Data Availability

The data analyzed in this study is subject to the following licenses/restrictions: this study was conducted using the UK Biobank data, which we are unable to share here, under Application Number 134551. Readers can apply the data from the UK Biobank website (www.ukbiobank.ac.uk). Descriptions for the variables can be found in https://biobank.ndph.ox.ac.uk/showcase/browse.cgi?id=-2&cd=category. Requests to access these datasets should be directed to www.ukbiobank.ac.uk.
