# Supplementary material for: Associations between the duration of outdoor sunlight exposure and the risk of developing 134 different diseases
Source: Front Public Health. 2026 Oct 26;14:1896657. doi: 10.3389/fpubh.2026.1896657 (PMC13745688; doi:10.3389/fpubh.2026.1896657)
Supplement: Supplementary file 1 [file Supplementary_file_1.docx]

**Associations between the duration of outdoor sunlight exposure and the risk of developing 134 different diseases**

**Sicheng Li^1^,** **Hanwen Zhou^2^, Wenpan Xian^3^, Yan Wang^1^**

^1^ Xiamen Cardiovascular Hospital of Xiamen University, School of Medicine, Xiamen University, Xiamen, Fujian, China

^2^ School of Public Health, Chongqing Medical University, Chongqing, China

^3^ Department of Stomatology, The First Affiliated Hospital of Xiamen University, School of Medicine, Xiamen University, Xiamen, Fujian, China

S. Li and H. Zhou contributed equally.

Correspondence:

**Sicheng Li**, Xiamen Cardiovascular Hospital of Xiamen University, School of Medicine, Xiamen University, No. 2999 Jinshan Road, Xiamen, 361006, Fujian, China. E-mail: [lisicheng1027@foxmail.com](mailto:lisicheng1027@foxmail.com)

**Wenpan Xian**, Department of Stomatology, The First Affiliated Hospital of Xiamen University, School of Medicine, Xiamen University, No. 2999 Jinshan Road, Xiamen, 361006, Fujian, China. Email: xianwenpan@qq.com

Contents

[Supplementary Texts	3](#_Toc236649029)

[Text S1 Covariates	3](#_Toc236649030)

[Text S2 Missing data and multiple imputation	4](#_Toc236649031)

[Text S3 Sensitivity analyses	4](#_Toc236649032)

[Text S4 Causal mediation analysis	5](#_Toc236649033)

[Text S5 Biological and phenotypic age	7](#_Toc236649034)

[Text S6 Mediators	8](#_Toc236649035)

[Supplementary Figures	10](#_Toc236649036)

[Figure S1 Folw chart of the study	10](#_Toc236649037)

[Figure S2 Descriptive analyses for sunlight exposure	11](#_Toc236649038)

[Figure S3 Distribution of missing data and its patterns	12](#_Toc236649039)

[Figure S4 Associations of sunlight with certain infectious and parasitic diseases	13](#_Toc236649040)

[Figure S5 Associations of sunlight with diseases of the blood involving the immune mechanism	14](#_Toc236649041)

[Figure S6 Associations of sunlight with endocrine, nutritional and metabolic diseases	16](#_Toc236649042)

[Figure S7 Associations of sunlight with mental and behavioural disorders	17](#_Toc236649043)

[Figure S8 Associations of sunlight with diseases of the eye and adnexa	19](#_Toc236649044)

[Figure S9 Associations of sunlight with diseases of the nervous system	21](#_Toc236649045)

[Figure S10 Associations of sunlight with diseases of the ear and mastoid process	21](#_Toc236649046)

[Figure S11 Associations of sunlight with diseases of the circulatory system	23](#_Toc236649047)

[Figure S12 Associations of sunlight with diseases of the respiratory system	25](#_Toc236649048)

[Figure S13 Associations of sunlight with diseases of the digestive system	27](#_Toc236649049)

[Figure S14 Associations of sunlight with diseases of the skin and subcutaneous tissue (SST)	29](#_Toc236649050)

[Figure S15 Associations of sunlight with diseases of the musculoskeletal system and connective tissue	31](#_Toc236649051)

[Figure S16 Associations of sunlight with diseases of the genitourinary system	33](#_Toc236649052)

[Figure S17 Associations of sunlight with neoplasms	35](#_Toc236649053)

[Supplementary Tables	36](#_Toc236649054)

[Table S1 Statistical description of continuous variables	36](#_Toc236649055)

[Table S2 Impact of missing data on population characteristics	36](#_Toc236649056)

[^Reference^	38](#_Toc236649057)

# Supplementary Texts

## Text S1 Covariates

The following variables were incorporated into the study in their original format: age (Field 21022, continuous), gender (Field 31, female, male), skin colour (Field 1717, black, brown, dark olive, fair, light olive, very fair), Townsend Deprivation Index (TDI, Field 22189, continuous), and location (Field 54, 22 assessment centers). Behavioral lifestyle variables included body mass index (BMI, Field 21001, continuous), metabolic equivalent of task (MET, Field 22040, continuous), sleep duration (Field 1160, continuous), smoking status (Field 20116, never, previous, current), drinking status (Field 1558, never, special occasions only, one to three times a month, once or twice a week, three or four times a week, daily or almost daily), and sun protection (Field 2267, do not go out in sunshine, never/rarely, sometimes, always, most of the time).

The following variables were incorporated into the study with simple processing for univariate only: education (Field 6138, with or without a college or university degree), employment status (Field 6142, employed, unemployed), vitamin D supplementation (Field 6155, yes, no).

The healthy diet index (HDI, continuous) was derived from participants’ intake of vegetables (Field 1289 and Field 1299), fruits (Field 1309 and Field 1319), fish (Field 1329 and Field 1339), processed meat (Field 1349), and non-processed red meat (Field 1369, Field 1379, and Field 1349), with each item scored as 1 for healthy and 0 for unhealthy, yielding an HDI range of 0 to 5, consistent with prior studies^1^**^,^**^2^.

Family history-related variables (yes, no) were derived from three variables: father’s diseases, mother’s diseases, and siblings’ diseases. For instance, a positive family history of hypertension is defined as having at least one parent or sibling with hypertension, whereas a negative family history of hypertension is defined as having no parent or sibling with hypertension. This variable is defined as missing for all other individuals.

Demographic characteristics were included as confounders because they were simultaneously associated with sunlight exposure and diseases. All these behavioral lifestyle variables are widely associated with diseases. Additionally, we use family histories of hypertension and diabetes to roughly depict genetic factors associated with all diseases. As MET^3^, BMI^4^, and sleep duration^5^ were associated with light exposure, they were included as potential mediators, and the remaining behavioral lifestyle and family history factors were included as effect modifiers.

Demographic characteristics were included as potential confounders because they may be associated with both outdoor sunlight exposure and disease risk. Behavioral lifestyle variables and family history were also considered because of their established associations with health outcomes. Vitamin D supplementation was treated as a baseline covariate rather than a mediator because the potential biological pathway of sunlight exposure is more directly represented by circulating vitamin D concentration, which was separately examined as a potential mediator in this study. Sun-protection measures were adjusted because they may modify the effective UV exposure received during a given duration spent outdoors and may also reflect health-related behaviors associated with disease risk. Although sun-protection behavior may partly respond to habitual outdoor exposure, its precise causal position cannot be determined from the available baseline data; therefore, its adjustment was intended primarily to reduce heterogeneity in effective UV exposure among individuals reporting similar outdoor durations.

## Text S2 Missing data and multiple imputation

The R package ***naniar*** and ***mice*** were used for visualization and multiple imputations of the missing data, respectively. In sensitivity analysis, we performed an imputation five times for the missing variables, using the predictive mean matching method for numerical variables, logistic regression imputation for binary variables, polytomous regression imputation for unordered categorical variables, and proportional odds model for unordered categorical variables^6^. After fitting five different Cox regression models, the function ***pool*** was used to integrate the results of multiple imputations.

**Text S3 Sensitivity analyses**

The reasons for conducting these sensitivity analyses are as follows: 1) excluding participants who developed specific diseases within two years after the baseline survey to reduce the risk of causal inversion; 2) utilizing multiple imputation techniques to address missing data; 3) further adjusting fine particulate matter as it may reduce light exposure through physical obstruction^7^ and are associated with health conditions^8^, potentially acting as a confounder; 4) further adjusting for some health-related environmental factors (including greenspace^9^ and noise^10^) associated with spending time outdoors, potentially acting as mediators; 5) further adjusting for self-reported health status to reflect individual disease status, as existing illnesses or sub-health status may lead to incident diseases and reduce participants’ willingness to go outside, potentially acting as a confounder; 6) further adjusting for self-reported medication for cholesterol, blood pressure or diabetes to consider current chronic diseases; 7) further adjusting for the baseline survey year to account for unmeasured confounders associated with time.

The following variables were incorporated into the sensitivity analyses in their original format: fine particulate matter (Field 24005, continuous), greenspace (Field 24500, continuous), noise (Field 24024, continuous), health status (Field 2178, excellent, good, fair, and poor), medication for cholesterol, blood pressure or diabetes (Fields 6177 and 6153, cholesterol lowering medication, blood pressure medication, insulin, and none of the above). Baseline survey year (2006, 2007, 2008, 2009, and 2010) is extracted solely from the year of the baseline survey date (Field 53).

**Text S4 Causal mediation analysis**

Assume that a given research sample with sample size N has one-dimensional exposure, mediator, and outcome variables. For each research subject $\boldsymbol{i\in1,2,\ldots,N}$ in the sample, let $\boldsymbol{A}_{\boldsymbol{i}}$ be the exposure it receives. $\boldsymbol{M}_{\boldsymbol{i}}$ is the mediator that is actually observed, and $\boldsymbol{C}_{\boldsymbol{i}}\boldsymbol{=}\boldsymbol{C}_{\boldsymbol{1}\boldsymbol{i}}\boldsymbol{,}\boldsymbol{C}_{\boldsymbol{2}\boldsymbol{i}}\boldsymbol{,}\boldsymbol{C}_{\boldsymbol{3}\boldsymbol{i}}\boldsymbol{,\ldots,}\boldsymbol{C}_{\boldsymbol{pi}}$ is a series of covariates that have been collected. $\boldsymbol{Y}_{\boldsymbol{i,a}}$ denotes the possible values of the outcome of the research subject $i$ in the case of exposure $\boldsymbol{A = a,}$ and $\boldsymbol{M}_{\boldsymbol{i,a}^{\boldsymbol{*}}}$ denotes the possible values of subject $i$'s mediator when exposure $\boldsymbol{A}\mathbf{=}\boldsymbol{a}^{\mathbf{*}}$, and it should be noted that $\boldsymbol{M}_{{\boldsymbol{i}\mathbf{,}\boldsymbol{a}}^{\mathbf{*}}}$ does not denote a certain fixed value, but rather denotes the mediator's potential value when $\boldsymbol{A=}\boldsymbol{a}^{\boldsymbol{*}}$. $\boldsymbol{Y}_{\boldsymbol{i,am}}$ denotes the possible values of subject $i$'s outcome when exposure $\boldsymbol{A=a}$ and the mediator $\boldsymbol{M = m}$. $\boldsymbol{Y}_{\boldsymbol{i}\mathbf{,}\boldsymbol{a}\boldsymbol{M}_{\boldsymbol{a}\mathbf{*}}}$ denotes the potential value of the mediated outcome of subject $i$ when exposure A = a and the mediator variable is at exposure $\boldsymbol{A=}\boldsymbol{a}^{\boldsymbol{*}}$. $\boldsymbol{Y}_{\boldsymbol{i}\mathbf{,}\boldsymbol{a}\boldsymbol{M}_{\boldsymbol{a}\mathbf{*}}}$ is the so-called nested counterfactual outcome, which takes into account both the role of exposure (denoted by a), and through the mediated potential variable $\boldsymbol{M}_{\boldsymbol{i,a}^{\boldsymbol{*}}}$ the effect of exposure on it, which takes into account the exposure-induced change in the mediator and the subsequent effect on the outcome. When exposure is dichotomous, there will be four nested counterfactuals $\boldsymbol{Y}_{\boldsymbol{i, 1}\boldsymbol{M}_{\boldsymbol{1}}}\boldsymbol{,}\boldsymbol{Y}_{\boldsymbol{i, 1}\boldsymbol{M}_{\boldsymbol{0}}}\boldsymbol{,}\boldsymbol{Y}_{\boldsymbol{i, 0}\boldsymbol{M}_{\boldsymbol{0}}}\boldsymbol{,}\boldsymbol{Y}_{\boldsymbol{i, 0}\boldsymbol{M}_{\boldsymbol{1}}}$ for each individual, and the target estimator will be defined by these nested counterfactual outcomes.

***Target estimator***

In mediation analysis, the most common target estimators include Controlled direct effect (CDE), Natural direct effect (NDE), and Natural indirect effect (NIE):

$$\boldsymbol{CDE=E}\left[ \boldsymbol{Y}_{\boldsymbol{am}}\boldsymbol{-}\boldsymbol{Y}_{\boldsymbol{a}^{\boldsymbol{*}}\boldsymbol{m}} \right]$$

$$\boldsymbol{NDE=E}\left[ \boldsymbol{Y}_{\boldsymbol{a}\boldsymbol{M}_{\boldsymbol{a}}}\boldsymbol{-}\boldsymbol{Y}_{\boldsymbol{a}^{\boldsymbol{*}}\boldsymbol{M}_{\boldsymbol{a}}} \right]$$

$$\boldsymbol{NIE=E}\left[ \boldsymbol{Y}_{\boldsymbol{a}\boldsymbol{M}_{\boldsymbol{a}}}\boldsymbol{-}\boldsymbol{Y}_{\boldsymbol{a}\boldsymbol{M}_{\boldsymbol{a}^{\boldsymbol{*}}}} \right]$$

The CDE is the mean difference in the value of the outcome of exposure compared to no exposure if all subjects are controlled by some means to the same level $\boldsymbol{m}$ of the mediator variable. The NIE is the mean difference value of the outcome if the exposures are all at level $\boldsymbol{a}$, but the mediator is at $\boldsymbol{M}_{\boldsymbol{a*}}$ and $\boldsymbol{M}_{\boldsymbol{a}}$, respectively. Similarly, the NDE is the mean difference in the outcome if the exposures are at $\boldsymbol{a}$ and $\boldsymbol{a}^{\boldsymbol{*}}$, but the mediator is at $\boldsymbol{M}_{\boldsymbol{a}}$.

***Statistical assumptions***

Within this theoretical framework of counterfactuals, the identification of causal mediating effects is based on the following four main assumptions:

***Hypothesis 1***: There is no uncorrected confounder of the association between the exposure and outcome. Given the set of covariates $\text{C}$, the exposure assignment mechanism is independent of the potential outcome.

$$\boldsymbol{Y}_{\boldsymbol{am}} \boldsymbol{\perp}\boldsymbol{A| C}$$

***Hypothesis 2***: There is no unadjusted confounder of the association between exposure and mediator. Given the set of covariates $\text{C}$, the exposure allocation mechanism is independent of the potential mediator taking.

$$\boldsymbol{M}_{\boldsymbol{a}} \boldsymbol{\perp}\boldsymbol{A|C}$$

***Hypothesis 3***: There is no unadjusted confounder between the mediator and outcome. Given the set of covariates $\text{C}$ and exposure $\boldsymbol{A=a}$, the assignment of the mediator is independent of the potential outcome.

$$\boldsymbol{Y}_{\boldsymbol{am}} \boldsymbol{\perp}\boldsymbol{M|}\left\{ \boldsymbol{A = a,C} \right\}$$

***Hypothesis 4***: Besides the three non-confounding assumptions of hypotheses 1 to 3 above, identifying mediating effects requires fulfilling the so-called “Cross-worlds independence” assumption. This condition holds when no confounders are influenced by the exposure between the mediator and the outcome.

$$\boldsymbol{Y}_{\boldsymbol{am}}\boldsymbol{\perp}\boldsymbol{M}_{\boldsymbol{a}^{\boldsymbol{*}}}\boldsymbol{|C}$$

After the above assumptions are met, causal mediation effects can be identified without making any assumptions about the model or parameter distributions, known as the mediation formula:

$$\boldsymbol{NDE=E}\left[ \boldsymbol{Y}_{\boldsymbol{a}\boldsymbol{M}_{\boldsymbol{a}^{\boldsymbol{*}}}}\boldsymbol{-}\boldsymbol{Y}_{\boldsymbol{a*}\boldsymbol{M}_{\boldsymbol{a}^{\boldsymbol{*}}}} \mid\boldsymbol{c} \right]\boldsymbol{=\sum E[Y\mid a,m,c]-E[Y\mid}\boldsymbol{a}^{\boldsymbol{*}}\boldsymbol{,m,c]P(m\mid}\boldsymbol{a}^{\boldsymbol{*}}\boldsymbol{,c)}$$

$$\boldsymbol{NIE=E[}\boldsymbol{Y}_{\boldsymbol{a}\boldsymbol{M}_{\boldsymbol{a}}}\boldsymbol{-}\boldsymbol{Y}_{\boldsymbol{a}\boldsymbol{M}_{\boldsymbol{a*}}}\boldsymbol{\mid c]=\sum E}\left[ \boldsymbol{Y} \mid\boldsymbol{a,m,c} \right]\boldsymbol{P}\left( \boldsymbol{m} \mid\boldsymbol{a,c} \right)\boldsymbol{-P}\left( \boldsymbol{m} \mid\boldsymbol{a}^{\boldsymbol{*}}\boldsymbol{,c} \right)$$

Technically, we incorporated mediator variables into the original Cox model to establish an exposure-mediator-outcome model. Expanding on this, we included all covariates to formulate an exposure-mediator linear model, facilitating the estimation of the mediation effect. When the lifestyle variable acts as a mediator, the exposure-mediator model does not control for it. We used the ***cmest*** function from the ***CMAverse*** R package for causal mediation analysis^11^.

**Text S5 Biological and phenotypic age**

Biological age was proposed by Klemera and Doubal^12^ based on eight biomarkers (C-reactive protein, serum creatinine, glycosylated hemoglobin, serum albumin, serum total cholesterol, serum urea nitrogen, serum alkaline phosphatase, and systolic blood pressure)^13^. R package ***bioage*** was used to calculate biological age in the analyses^14^. The values ***j*** and ***i*** represent the number of biomarkers and samples, respectively. The values ***k***, ***q***, and ***s*** are the regression slope, intercept, and root means squared error of a biomarker regressed on chronological age, respectively. The value $\boldsymbol{r}_{\boldsymbol{j}}^{\boldsymbol{2}}$ represents the variance explained by regression of chronological age on biomarkers. All the remaining covariates and variables used in the flow chart and calculating biological age are shown in.

$$\begin{aligned} \boldsymbol{BA}_{\boldsymbol{E}}\boldsymbol{=}\frac{\sum_{\boldsymbol{j=1}}^{\boldsymbol{m}} \left( \boldsymbol{x}_{\boldsymbol{j}}\boldsymbol{-}\boldsymbol{q}_{\boldsymbol{j}} \right)\left( \frac{\boldsymbol{k}_{\boldsymbol{j}}}{\boldsymbol{s}_{\boldsymbol{j}}^{\boldsymbol{2}}} \right)}{\sum_{\boldsymbol{j=1}}^{\boldsymbol{m}} \left( \frac{\boldsymbol{k}_{\boldsymbol{j}}}{\boldsymbol{s}_{\boldsymbol{j}}} \right)^{\boldsymbol{2}}}\boldsymbol{\#}\left( \mathbf{1} \right) \end{aligned}$$

$$\begin{aligned} \boldsymbol{r}_{\boldsymbol{char}}\boldsymbol{=}\frac{\sum_{\boldsymbol{j=1}}^{\boldsymbol{m}} \frac{\boldsymbol{r}_{\boldsymbol{j}}^{\boldsymbol{2}}}{\sqrt{\boldsymbol{1-}\boldsymbol{r}_{\boldsymbol{j}}^{\boldsymbol{2}}}}}{\sum_{\boldsymbol{j=1}}^{\boldsymbol{m}} \frac{\boldsymbol{r}_{\boldsymbol{j}}}{\sqrt{\boldsymbol{1-}\boldsymbol{r}_{\boldsymbol{j}}^{\boldsymbol{2}}}}}\boldsymbol{\#}\left( \boldsymbol{2} \right) \end{aligned}$$

$$\begin{aligned} \boldsymbol{S}_{\boldsymbol{BA}}^{\boldsymbol{2}}\boldsymbol{=}\frac{\sum_{\boldsymbol{j=1}}^{\boldsymbol{n}} \left( \left( \boldsymbol{BA}_{\boldsymbol{Ei}}\boldsymbol{-}\boldsymbol{CA}_{\boldsymbol{i}} \right)\boldsymbol{-}\frac{\sum_{\boldsymbol{i=1}}^{\boldsymbol{n}} \left( \boldsymbol{BA}_{\boldsymbol{Ei}}\boldsymbol{-}\boldsymbol{CA}_{\boldsymbol{i}} \right)}{\boldsymbol{n}} \right)^{\boldsymbol{2}}}{\boldsymbol{n}}\boldsymbol{-}\left( \frac{\boldsymbol{1-}\boldsymbol{r}_{\boldsymbol{char}}^{\boldsymbol{2}}}{\boldsymbol{r}_{\boldsymbol{char}}^{\boldsymbol{2}}} \right)\left( \frac{\left( \boldsymbol{CA}_{\boldsymbol{max}}\boldsymbol{-}\boldsymbol{CA}_{\boldsymbol{min}} \right)^{\boldsymbol{2}}}{\boldsymbol{12}\boldsymbol{m}} \right)\boldsymbol{\#}\left( \boldsymbol{3} \right) \end{aligned}$$

$$\begin{aligned} \boldsymbol{Biological age=}\frac{\sum_{\boldsymbol{j=1}}^{\boldsymbol{m}} \left( \boldsymbol{x}_{\boldsymbol{j}}\boldsymbol{-}\boldsymbol{q}_{\boldsymbol{j}} \right)\left( \frac{\boldsymbol{k}_{\boldsymbol{j}}}{\boldsymbol{s}_{\boldsymbol{j}}^{\boldsymbol{2}}} \right)\boldsymbol{+}\frac{\boldsymbol{CA}}{\boldsymbol{s}_{\boldsymbol{BA}}^{\boldsymbol{2}}}}{\sum_{\boldsymbol{j=1}}^{\boldsymbol{m}} \left( \frac{\boldsymbol{k}_{\boldsymbol{j}}}{\boldsymbol{s}_{\boldsymbol{j}}} \right)^{\boldsymbol{2}}\boldsymbol{+}\frac{\boldsymbol{1}}{\boldsymbol{s}_{\boldsymbol{BA}}^{\boldsymbol{2}}}}\boldsymbol{\#}\left( \boldsymbol{4} \right) \end{aligned}$$

Phenotypic age was proposed by Levine based on nine agingrelated variables using the following formula^15^:

$$\begin{aligned} \boldsymbol{Phenotypic age=}\boldsymbol{141.50225}\boldsymbol{+}\frac{\boldsymbol{ln}\left( \boldsymbol{-0.00553\times}\boldsymbol{ln}\left( \boldsymbol{1-MortalityRis}\boldsymbol{k} \right) \right)}{\boldsymbol{0.090165}}\boldsymbol{\#}\left( \boldsymbol{5} \right) \end{aligned}$$

$$\begin{aligned} \boldsymbol{MortalityRis}\boldsymbol{k}\boldsymbol{=}\boldsymbol{1-}\boldsymbol{exp}\left( \frac{\boldsymbol{-1.51714\times}\boldsymbol{exp}\left( \boldsymbol{xb} \right)}{\boldsymbol{0.0076927}} \right)\boldsymbol{\#}\left( \boldsymbol{6} \right) \end{aligned}$$

where xb= -19.907 − 0.0336 × albumin + 0.0095 × creatinine + 0.1953 × glucose + 0.0954× ln(C-reactive protein) − 0.0120 × lymphocyte percentage + 0.0268 × mean corpuscular volume + 0.3306 × red blood cell distribution width + 0.00188 × alkaline phosphatase + 0.0554 × white blood cell count + 0.0804 × chronological age.

**Text S6 Mediators**

All the remaining mediators were continuous and incorporated into the study in their original format. Inflammation indicators included C-reactive protein (Field 30710), white blood cell count (Field 30000), neutrophil count (Field 30140), platelet count (Field 30080), lymphocyte count (Field 30120), systemic immune-inflammation index (platelet×neutrophil÷lymphocyte), direct bilirubin (Field 30660), and total bilirubin (Field 30840). Lipids included triglyceride (Field 30870), cholesterol (Field 26037), high-density lipoprotein (Field 30760), low-density lipoprotein (Field 30780), apolipoprotein A (Field 30630), apolipoprotein B (Field 30640), and lipoprotein A (Field 30790). General blood biochemical indicators included calcium (Field 30680), vitamin D (Field 30890), creatinine (Field 30700), urea (Field 30670), glucose (Field 23470), alkaline phosphatase (Field 30610), albumin (Field 30600), hemoglobin A_1c_ (Field 30750), Insulin-like growth factors-1 (Field 30770), gamma-glutamyl transferase (Field 30730), aspartate aminotransferase (Field 30650), and alanine aminotransferase (Field 30620). Lifestyle factors included BMI, MET, HDI, and sleep duration.

# Supplementary Figures

### Figure S1 Flow chart of the study

### Figure S2 Descriptive analyses for sunlight exposure

### Figure S3 Distribution of missing data and its patterns

This figure includes only the top 10 variables characterized by a substantial number of missing values, encompassing exposure, outcome, and covariate variables.

In the lower-left corner, we display the types of variables with missing data and the corresponding count of affected individuals. Meanwhile, in the lower-right corner, we present the various missing patterns, which indicate the presence of specific variables among the excluded individuals. Additionally, in the upper-right corner, we show the precise number of individuals conforming to each missing pattern.

Abbreviations: HDI: Healthy diet index; MET: Metabolic equivalent of task.

### Figure S4 Associations of sunlight with certain infectious and parasitic diseases

### Figure S5 Associations of sunlight with diseases of the blood involving the immune mechanism

### Figure S6 Associations of sunlight with endocrine, nutritional and metabolic diseases

### Figure S7 Associations of sunlight with mental and behavioural disorders

### Figure S8 Associations of sunlight with diseases of the eye and adnexa

### Figure S9 Associations of sunlight with diseases of the nervous system

### Figure S10 Associations of sunlight with diseases of the ear and mastoid process

### Figure S11 Associations of sunlight with diseases of the circulatory system

### Figure S12 Associations of sunlight with diseases of the respiratory system

### Figure S13 Associations of sunlight with diseases of the digestive system

### Figure S14 Associations of sunlight with diseases of the skin and subcutaneous tissue (SST)

### Figure S15 Associations of sunlight with diseases of the musculoskeletal system and connective tissue

### Figure S16 Associations of sunlight with diseases of the genitourinary system

### Figure S17 Associations of sunlight with neoplasms

# Supplementary Tables

| **Table S1 Statistical description of continuous variables** | | | | | | | | |
| --- | --- | --- | --- | --- | --- | --- | --- | --- |
| **Variable** | **Mean** | **SD** | **IQR** | **Max** | ***P*_75_** | **Median** | ***P*_25_** | **Min** |
| Summer light ***** | 3.3 | 1.8 | 2 | 8 | 4 | 3 | 2 | 0 |
| Winter light | 1.5 | 1.0 | 1 | 4 | 2 | 1 | 1 | 0 |
| Average light | 2.4 | 1.2 | 1.5 | 6 | 3 | 2 | 1.5 | 0 |

Abbreviations: SD: standard deviation; IQR: interquartile range; ***P***_75_: upper quartile; ***P***_25_: lower quartile.

| **Table S2 Impact of missing data on population characteristics** | | | |
| --- | --- | --- | --- |
| **Characteristic** | **Complete ***  (N = 281,282) | **Entire †**  (N = 457,061) | ***P*** **‡** |
| **DEMOGRAPHIC CHARACTERISTICS** |  |  |  |
| Age | 55.81 (8.07) **§** | 56.46 (8.08) | <0.001 |
| TDI | -1.60 (2.91) | -1.35 (3.07) | <0.001 |
| Gender (Female) | 156,920 (55.79%) | 259,643 (56.81%) | <0.001 |
| Work (Employed) | 180,538 (64.18%) | 273,785 (60.25%) | <0.001 |
| Education (with a college or university degree) | 111,767 (39.73%) | 153,892 (34.36%) | <0.001 |
| Location |  |  | 0.054 |
| England | 249,092 (88.56%) | 405,424 (88.70%) |  |
| Scotland | 20,658 (7.34%) | 32,882 (7.19%) |  |
| Wales | 11,532 (4.10%) | 18,755 (4.10%) |  |
| Skin colour |  |  | <0.001 |
| Black | 1,296 (0.46%) | 3,272 (0.73%) |  |
| Brown | 6,370 (2.26%) | 12,900 (2.87%) |  |
| Dark olive | 4,888 (1.74%) | 7,834 (1.74%) |  |
| Fair | 191,962 (68.25%) | 306,886 (68.24%) |  |
| Light olive | 54,442 (19.35%) | 83,124 (18.48%) |  |
| Very fair | 22,324 (7.94%) | 35,702 (7.94%) |  |
| **LIFESTYLE** |  |  |  |
| BMI | 27.09 (4.66) | 27.37 (4.82) | <0.001 |
| MET | 2,452.35 (2,391.01) | 2,455.56 (2,423.82) | 0.002 |
| Sleep duration | 7.17 (1.04) | 7.15 (1.10) | <0.001 |
| HEI | 2.91 (1.28) | 2.88 (1.28) | <0.001 |
| Vitamin D supplement (Yes) | 11,524 (4.10%) | 18,427 (4.09%) | >0.9 |
| Smoke status |  |  | <0.001 |
| Never | 159,394 (56.67%) | 252,422 (55.55%) |  |
| Previous | 96,328 (34.25%) | 156,168 (34.37%) |  |
| Current | 25,560 (9.09%) | 45,804 (10.08%) |  |
| Alcohol status |  |  | <0.001 |
| Never | 18,319 (6.51%) | 36,285 (7.96%) |  |
| Special occasions only | 28,076 (9.98%) | 52,633 (11.55%) |  |
| One to three times a month | 30,834 (10.96%) | 51,117 (11.22%) |  |
| Once or twice a week | 72,289 (25.70%) | 116,912 (25.66%) |  |
| Three or four times a week | 70,074 (24.91%) | 105,824 (23.23%) |  |
| Daily or almost daily | 61,690 (21.93%) | 92,846 (20.38%) |  |
| Sun protection |  |  | <0.001 |
| Never/rarely | 23,609 (8.39%) | 43,339 (9.60%) |  |
| Sometimes | 91,145 (32.40%) | 148,684 (32.94%) |  |
| Always | 59,399 (21.12%) | 93,834 (20.79%) |  |
| Most of the time | 105,839 (37.63%) | 162,649 (36.03%) |  |
| Do not go out in sunshine | 1,290 (0.46%) | 2,860 (0.63%) |  |
| **EXPOSURE** |  |  |  |
| Summer light | 3.26 (1.81) | 3.33 (1.84) | <0.001 |
| Winter light | 1.49 (0.98) | 1.51 (1.01) | 0.001 |
| Average light | 2.38 (1.25) | 2.42 (1.27) | <0.001 |
| **FAMILY HISTORY** |  |  | <0.001 |
| Family history of diabetes (Yes) | 64,562 (22.95%) | 98,563 (25.46%) |  |
| Family history of hypertension (Yes) | 143,271 (50.94%) | 219,678 (54.12%) |  |
| **POTENTIAL MECHANISM INDICATORS** |  |  |  |
| BA, year | 54.38 (14.65) | 55.62 (14.90) | <0.001 |
| Phenoage, year | 50.95 (10.04) | 51.91 (10.20) | <0.001 |
| Telomere | 0.03 (1.00) | 0.01 (1.00) | <0.001 |
| CRP (lg), mg/L | 0.25 (1.05) | 0.32 (1.07) | <0.001 |
| Neutrophill, 10*9 cells/L | 4.17 (1.39) | 4.22 (1.42) | <0.001 |
| WBC, 10*9 cells/L | 6.80 (2.13) | 6.87 (2.14) | <0.001 |
| SII | 593.08 (370.51) | 599.77 (382.80) | <0.001 |
| Calcium, mmol/L | 2.38 (0.09) | 2.38 (0.09) | 0.037 |
| Vitamin D, nmol/L | 49.08 (20.99) | 48.31 (21.00) | <0.001 |

***:** The complete population is the remaining population after excluding missing data for the entire population, i.e. the population finally included in the analysis.

**†:** The entire population refers to the population remaining after going through the exclusion criteria in the flowchart (**Figure 1**) without excluding missing data.

**‡:** Pearson’s Chi-squared test was used for categorical variables, and the Wilcoxon rank sum test was used for continuous variables.

**§:** Continuous variables are presented as mean (standard deviation), and discrete variables are presented as n(%).

# Reference

1.	Wang M, Zhou T, Song Y, et al. Joint exposure to various ambient air pollutants and incident heart failure: a prospective analysis in UK Biobank. *European heart journal* 2021; **42**(16): 1582-91.

2.	Xu H, Xu H, Wu J, et al. Ambient air pollution exposure, plasma metabolomic markers, and risk of type 2 diabetes: A prospective cohort study. *Journal of hazardous materials* 2024; **463**: 132844.

3.	Zheng C, Feng J, Huang W, Wong SH-S. Associations between weather conditions and physical activity and sedentary time in children and adolescents: A systematic review and meta-analysis. *Health & Place* 2021; **69**: 102546.

4.	Klinedinst BS, Meier NF, Larsen B, et al. Walking in the Light: How History of Physical Activity, Sunlight, and Vitamin D Account for Body Fat-A UK Biobank Study. *Obesity (Silver Spring)* 2020; **28**(8): 1428-37.

5.	Burns AC, Saxena R, Vetter C, Phillips AJK, Lane JM, Cain SW. Time spent in outdoor light is associated with mood, sleep, and circadian rhythm-related outcomes: A cross-sectional and longitudinal study in over 400,000 UK Biobank participants. *J Affect Disord* 2021; **295**: 347-52.

6.	Buuren Sv, Groothuis-Oudshoorn K, Vink G, et al. mice: Multivariate Imputation by Chained Equations. 2021. <https://cran.r-project.org/web/packages/mice/>.

7.	Ma LZ, Ma YH, Ou YN, et al. Time spent in outdoor light is associated with the risk of dementia: a prospective cohort study of 362094 participants. *BMC Med* 2022; **20**(1): 132.

8.	Nan N, Yan Z, Zhang Y, Chen R, Qin G, Sang N. Overview of PM2.5 and health outcomes: Focusing on components, sources, and pollutant mixture co-exposure. *Chemosphere* 2023; **323**: 138181.

9.	Yang B-Y, Zhao T, Hu L-X, et al. Greenspace and human health: An umbrella review. *The Innovation* 2021; **2**(4).

10.	Chen X, Liu M, Zuo L, et al. Environmental noise exposure and health outcomes: an umbrella review of systematic reviews and meta-analysis. *European Journal of Public Health* 2023; **33**(4): 725-31.

11.	Shi B, Choirat C, Coull BA, VanderWeele TJ, Valeri L. CMAverse: A Suite of Functions for Reproducible Causal Mediation Analyses. *Epidemiology* 2021; **32**(5): e20-e2.

12.	Klemera P, Doubal S. A new approach to the concept and computation of biological age. *Mech Ageing Dev* 2006; **127**(3): 240-8.

13.	Chen L, Zhao Y, Liu F, et al. Biological aging mediates the associations between urinary metals and osteoarthritis among U.S. adults. *BMC Medicine* 2022; **20**(1): 207.

14.	Parker DC, Bartlett BN, Cohen HJ, et al. Association of Blood Chemistry Quantifications of Biological Aging With Disability and Mortality in Older Adults. *The Journals of Gerontology: Series A* 2019; **75**(9): 1671-9.

15.	Levine ME, Lu AT, Quach A, et al. An epigenetic biomarker of aging for lifespan and healthspan. *Aging (Albany NY)* 2018; **10**(4): 573-91.
